# The added value of mammography in different age-groups of women with and without *BRCA* mutation screened with breast MRI

**DOI:** 10.1186/s13058-018-1019-6

**Published:** 2018-08-03

**Authors:** Suzan Vreemann, Jan C. M. van Zelst, Margrethe Schlooz-Vries, Peter Bult, Nicoline Hoogerbrugge, Nico Karssemeijer, Albert Gubern-Mérida, Ritse M. Mann

**Affiliations:** 1 Department of Radiology and Nuclear Medicine, Radboud University Medical Center, Geert Grooteplein 10, 6525 GA Nijmegen, the Netherlands; 2 Department of Surgery, Radboud University Medical Center, Nijmegen, the Netherlands; 3 Department of Pathology, Radboud University Medical Center, Nijmegen, the Netherlands; 4 Department of Human Genetics, Radboud University Medical Center, Nijmegen, the Netherlands

**Keywords:** Mammography, Breast MRI, High-risk screening, Age-categories, Screen-detected breast cancer, False positives

## Abstract

**Background:**

Breast magnetic resonance imaging (MRI) is the most sensitive imaging method for breast cancer detection and is therefore offered as a screening technique to women at increased risk of developing breast cancer. However, mammography is currently added from the age of 30 without proven benefits. The purpose of this study is to investigate the added cancer detection of mammography when breast MRI is available, focusing on the value in women with and without *BRCA* mutation, and in the age groups above and below 50 years.

**Methods:**

This retrospective single-center study evaluated 6553 screening rounds in 2026 women at increased risk of breast cancer (1 January 2003 to 1 January 2014). Risk category (*BRCA* mutation versus others at increased risk of breast cancer), age at examination, recall, biopsy, and histopathological diagnosis were recorded. Cancer yield, false positive recall rate (FPR), and false positive biopsy rate (FPB) were calculated using generalized estimating equations for separate age categories (< 40, 40–50, 50–60, ≥ 60 years). Numbers of screens needed to detect an additional breast cancer with mammography (NSN) were calculated for the subgroups.

**Results:**

Of a total of 125 screen-detected breast cancers, 112 were detected by MRI and 66 by mammography: 13 cancers were solely detected by mammography, including 8 cases of ductal carcinoma in situ. In *BRCA* mutation carriers, 3 of 61 cancers were detected only on mammography, while in other women 10 of 64 cases were detected with mammography alone. While 77% of mammography-detected-only cancers were detected in women ≥ 50 years of age, mammography also added more to the FPR in these women. Below 50 years the number of mammographic examinations needed to find an MRI-occult cancer was 1427.

**Conclusions:**

Mammography is of limited added value in terms of cancer detection when breast MRI is available for women of all ages who are at increased risk. While the benefit appears slightly larger in women over 50 years of age without *BRCA* mutation, there is also a substantial increase in false positive findings in these women.

## Background

Mammography-based screening for breast cancer reduces breast cancer-related mortality in the general female population [1]. However, in women at increased risk (e.g. those with a germline mutation in the *BRCA1* or *BRCA2* genes) biennial mammographic screening is insufficient due to low sensitivity and high rates of interval cancers [2–5]. Consequently, these women who have a higher-than-average lifetime risk of breast cancer (approximately ≥ 20–25% life time risk (LTR)) are invited to intensified screening programs [6, 7], consisting of dynamic contrast-enhanced magnetic resonance imaging (DCE-MRI) and mammography. The sensitivity and specificity of these screening programs have been reported to be as high as 97% and 98%, respectively [4, 8–12].

Recent studies question the added cancer detection of mammography in this population, especially in *BRCA* mutation carriers [13]. In the study of Kuhl et al. [10], MRI proved to be the most important contributor to stage reduction. Although these results show the superiority of breast MRI compared to mammography for the detection of cancers, routine mammography is currently recommended for all women, even at a relatively young age. Various authors have proposed to cancel mammographic screening in young women also screened with breast MRI, especially in *BRCA1* mutation carriers. In these *BRCA1* mutation carriers, the mammographic sensitivity is exceedingly low, reported as low as 35% [14]. This is believed to be caused not only by the on-average dense breasts of these women, but also by the mammographic benign-like features of *BRCA1*-associated cancers [15, 16]. Berrington de Gonzalez et al. reported that there is little to no benefit of mammographic screening under the age of 35 [17]. Additionally, concerns are raised about the risk of radiation-induced cancers in these women, as *BRCA* mutation carriers have increased susceptibility to radiation [17, 18].

Although guidelines may vary per country, mammographic screening in *BRCA* mutation carriers is advised from the age of 30 years [6, 7]. However, the actual benefits in terms of tumor detection of the addition of mammography at such a young age are still unclear. Furthermore, additional findings on the mammogram might lead to an increase in false positive recalls in the screening program.

Hence, there is a clinical need to find an optimal regimen for intensified screening programs to prevent unnecessary recalls, biopsies, and radiation exposure. The purpose of this study is to evaluate the added cancer detection and false positive rates with mammography when breast MRI is available in a population of women at increased risk of developing breast cancer. Differences in the complementary value of mammography in women below and above 50 years of age, and in *BRCA* mutation carriers versus others at increased risk of breast cancer were assessed.

## Methods

This retrospective study was approved by our local institutional review board and the requirement for informed consent was waived.

### Screening program

The increased risk screening program was evaluated for the period 1 January 2003 until 1 January 2014. The program starts at age 25 years for *BRCA* mutation carriers, who undergo yearly MRI. At the age of 30 years a yearly mammography is added. Women with an LTR of ≥ 20–25% are screened from the start with mammography and MRI; starting ages differ by the reason for screening [19]. Furthermore, women may have been enrolled in the program at a later point in time after detection of a specific factor that increases their personal risk. We previously reported on the overall screening performance in this cohort [20].

### Case selection

The local database was searched to identify all screening MRI and mammography examinations. Women were included when an MRI examination was considered a screening examination (inquiry at the radiology department was for screening purposes in asymptomatic women). Women were excluded when no mammography was performed within 6 months of the screening MRI. Risk category, age, screening tests performed, eventual recall for workup of screen-detected abnormalities and histopathological diagnosis were recorded when available.

### Image acquisition

MRI acquisition and protocols varied over time and have previously been reported in detail [21]. In short: examinations were performed on either a 1.5 or 3.0 Tesla Siemens scanner (Magnetom Avanto, Magnetom Sonata, Magnetom symphony or Magnetom Trio) using a dedicated bilateral breast coil. Patients were imaged in the prone position. A transverse or coronal three-dimensional T1-weighted gradient-echo dynamic sequence was performed before contrast agent administration followed by four or five post-contrast sequences. Various gadolinium chelates were used as a contrast agent, administered at a dose of 0.1 mmol/kg or 0.2 mmol/kg body weight using a power injector (Medrad, Warrendale, PA, USA) at a flow rate of 2.5 mL/s, followed by a saline flush. Premenopausal women were scheduled in the 6th to 12th day of their menstrual cycle.

Mammograms were obtained in two directions (medio-lateral oblique and cranio-caudal) with a full-field digital mammography machine (GE Senograph 2000 or GE Senograph DS, GE, Fairfield, CT, USA). Additional views and spot compression views were performed at request of the evaluating radiologist.

### Image interpretation

The Breast Imaging Reporting and Data system (BI-RADS) [22, 23] was used for evaluation. All examinations were evaluated by one of eight breast radiologists with experience ranging from 0.5 to 23 years after certification. Images were reported using a dedicated breast MRI workstation (versions of DynaCAD, Invivo, Philips, Best, the Netherlands). Mammograms were evaluated together with MRI examinations when these examinations were acquired the same day. In general, biopsies were performed for lesions classified as BI-RADS 4 and 5, and a subset of lesions classified as BI-RADS 3. The remainder of BI-RADS 3 lesions underwent short-term follow up.

### Ground truth

For BI-RADS 3 lesions with a short-term follow-up recommendation, at least 1 year of clinical follow up was required to confirm benignity. A cross-computer search of our pathology records was performed to identify all biopsies performed. We subsequently analyzed if the biopsy was triggered by screening results or whether the woman presented with symptoms. To ensure detection of all cancers, the database was also linked to the nationwide population-based Netherlands Cancer Registry (NCR).

### Data analysis

Pathology results were grouped into malignant (in situ, invasive, and metastatic cancer) and benign lesions (all other findings). Only screen-detected cancers were investigated, which were defined as cancers diagnosed after diagnostic workup initiated by screening results. We separated screen-detected cancers by mammography, MRI, or both based on radiological reports of the respective modalities (or report sections when mammograms and MRI were reported simultaneously).

Cancer yield, false positive recall rate (FPR) and false positive biopsy rate (FPB) for mammography, MRI, and the combination were calculated. Cancer yield was defined as the number of screen-detected cancers per 1000 screening rounds. An FPR or FPB was defined as a woman who was recalled/biopsied and was considered disease-free after workup and/or after at least 1 year of clinical follow up. The FPR/FPB were defined as the number of FPRs/FPBs per 1000 screening rounds.

Two risk categories were evaluated (*BRCA* mutation carriers and all others). The *BRCA* mutation carriers group also included first-degree untested relatives. Examinations were grouped into four age categories to investigate the influence of age (< 40, 40–50, 50–60, ≥ 60 years).

### Statistical analysis

Descriptive statistics were extracted. The chi-square (χ^2^) test was applied to compare differences between groups in demographics, in proportion of breast cancer, invasive cancer, ductal carcinoma in situ (DCIS), tumor grade, and false positives. Chi-square trend-tests were performed to investigate the distribution of parameters across age categories. Repeated screening results were summarized to form binomial counts for each woman to estimate cancer yield, FPR, and FPB. For each woman, the number of true-positive and true-negative screens per modality, and the number of screening visits with or without breast cancer detected were counted. In this way, binomial counts per modality were calculated and analyzed. As the dependent variable was assumed to follow a binomial distribution, generalized estimating equations (GEE) were applied. The binomial proportions were modeled and conducted separately for cancer yield, FPR, and FPB, using a compound symmetry correlation structure. The analysis was conducted separately for each age category, modality, and risk category. After applying the Bonferroni correction, a two-sided *p* value of 0.013 was considered statistically significant. The number of mammography screens needed (NSN) to detect one breast cancer that was missed by MRI was calculated by dividing the number of mammography screens performed by the number of breast cancers detected by mammography alone. All statistics were performed using SPSS (version 22, SPSS Inc., Chicago, IL, USA).

## Results

### Study population

Final analysis included 2026 women with 6553 screening rounds (Table 1 and Table 2): 125 screen-detected cancers were identified of which 13 and 59 were only detected by mammography or MRI, respectively (*p* < 0.001). In total, 112 cancers were seen on MRI and 66 on mammography. Overall, no significant difference was found between tumor grade of cancers detected by mammography or MRI (*p* = 0.193). Mammography detected a significantly larger proportion of pure DCIS (16/66 (24%) and 15/112 (13%) for mammography or MRI, respectively, *p* < 0.001). We did not observe a difference in the grade of DCIS detected with mammography or MRI (*p* = 0.436).

**Table 1:** Demographic data and risk profile

	All women	*BRCA* mutation carriers^a^	Others at increased risk^b^
Number	2026	744	1282
Age			
Mean	44.7	40.4	47.2
SD	11.7	11.0	11.3
Median	44	39	47
Range	21–91	23–75	21–91
Number of cancers	125	61	64
Number of false positive recalls	502	165	337
Number of false positive biopsies	331	117	214

^a^*BRCA* mutation carriers include 454 *BRCA1* mutation carriers and 290 *BRCA2* mutation carriers

^b^Others at increased risk include 561 women with a family history of breast cancer, 515 women with a personal history of breast cancer, and 206 others

**Table 2:** Population and breast cancer characteristics in the cohort

	Age < 40 years	Age 40–50 years	Age 50–60 years	Age ≥ 60 years	Overall	*p* value^a^
Women (*N*)						
*BRCA*	388	258	182	75	903	<0.001
Others	329	504	482	273	1588	0.014
Overall	717	762	664	348	2491	<0.001
Exams (N)						
*BRCA*	1113	737	568	190	2608	<0.001
Others	716	1313	1265	651	3945	0.046
Overall	1829	2050	1833	841	6553	<0.001
BC (N)						
Mammography	13	13	25	15	66	0.253
Mammography only	1	2	8	2	13	0.202
MRI	25	30	37	20	112	0.697
MRI only	13	19	20	7	59	0.254
Overall	26	32	45	22	125	0.963
Invasive tumor (*N*)						
Mammography	13	9	16	12	50	0.771
Mammography only	1	0	4	0	5	0.822
MRI	24	25	30	18	97	0.496
MRI only	12	16	18	6	52	0.253
Overall	25	25	34	18	102	0.540
DCIS (*N*)						
Mammography	0	4	9	3	16	0.073
Mammography only	0	2	5	1	8	0.281
MRI	1	5	7	2	15	0.036
MRI only	1	3	2	1	7	0.848
Overall	1	7	11	4	23	0.164
Tumor grade of all cancers (invasive and in situ) (*N*)						
Grade 1						
Mammography	1	1	4	1	7	0.536
Mammography only	0	0	1	0	1	0.655
MRI	3	4	5	5	17	0.384
MRI only	2	3	2	4	11	0.442
Grade 2						
Mammography	1	5	7	4	17	0.171
Mammography only	0	1	2	1	4	0.317
MRI	3	10	17	6	36	0.170
MRI only	2	6	12	3	23	0.335
Grade 3						
Mammography	11	7	11	6	35	0.339
Mammography only	1	1	2	1	5	0.822
MRI	17	15	15	5	52	0.010
MRI only	7	9	6	0	22	0.009
Missing	2	1	3	4	10	0.197
FPR (*N*)						
Mammography	63	75	55	22	215	<0.001
Mammography only	22	38	28	15	103	0.115
MRI	159	143	72	25	399	<0.001
MRI only	118	106	45	18	287	<0.001
Overall	181	181	100	40	502	<0.001
FPB (*N*)						
Mammography	35	46	32	9	122	<0.001
Mammography only	6	15	11	3	35	0.258
MRI	114	113	51	18	296	<0.001
MRI only	85	82	30	12	209	<0.001
Overall	120	128	62	21	331	<0.001

*BC* breast cancer (invasive cancer and ductal carcinoma in situ (DCIS)), *MRI* magnetic resonance imaging, *FPR* false positive recall, *FPB* false positive biopsy

^a^Chi-square test for trend was performed for the fraction of the overall population

### Mammography-detected breast cancers

The majority of cancers detected only with mammography consisted of pure DCIS (pTis) (8/13, 62%, Table 3). Most women who were diagnosed with pure DCIS were ≥ 50 years of age (6/8, 75%, Table 3). The remaining five women with an invasive cancer detected only at mammography were aged 35, 53, 54, 55, and 56 years, respectively. Overall, cancer detection with mammography only was higher in women ≥ 50 years old, than in those below 50, though this was not significant (3/58 vs. 10/67, *p* = 0.07). All pure mammography-detected breast cancers were detected in follow-up rounds. The NSN for the overall population and the defined subgroups are presented in Table 4. There was no cancer that was not reported by MRI in the first rounds of screening, making an estimate of NSN not applicable. Our results show that the NSN was highest in the lowest age categories. Whether there is a difference in age groups between women with a proven *BRCA* mutation and women without is difficult to determine, since we did not observe only mammographically detected breast cancers in *BRCA* mutation carriers under 50 years of age, but overall the added cancer detection in *BRCA* mutation carriers was slightly lower than in other women at increased risk (3/61 vs. 10/64, *p* = 0.05).

**Table 3:** Breast cancers detected solely by mammography

Number	Risk category	Ipsi/ contra^b^	Age	Tumor type	Tumor size^a^	Tumor grade	ER-status	PR- status	H2N-status	Nodal status	1st round versus FU
1	*BRCA1*	N/A	50	DCIS	6	2	–	–	–	0	FU
2	Family	N/A	43	DCIS	7	2	–	–	–	0	FU
3	Family	N/A	48	DCIS	–	3	–	–	–	0	FU
4	Personal	Ipsi	55	DCIS	23	–	–	–	–	0	FU
5	Personal	Contra	58	DCIS	–	–	–	–	–	0	FU
6	Personal	Contra	69	DCIS	6	2	–	–	–	0	FU
7	Other	N/A	61	DCIS	21	3	–	–	–	–	FU
8	Personal	Contra	55	DCIS	–	3	–	–	–	0	FU
9	*BRCA2*	N/A	57	DCIS^1^	6	–	Positive	Positive	Negative	0	FU
10	*BRCA1*	N/A	56	IDC	8	3	Positive	Positive	Negative	0	FU
11	Family	N/A	35	IDC	4	3	Positive	Positive	–	0	FU
12	Family	N/A	53	Tubular	3	1	Positive	Positive	Negative	0	FU
13	Other	N/A	54	ILC	23	2	Positive	Positive	Negative	1mi	FU

The symbol “–” indicates not available

*N/A* not applicable, *ipsi* ipsilateral, *contra* contralateral, *ER* estrogen receptor, *PR* progesterone receptor, *DCIS* ductal carcinoma in situ, *IDC* invasive ductal carcinoma, *ILC* invasive lobular carcinoma, *FU* follow up

^a^Pathological tumor size (in mm), in case of multi-centric tumors (case 13) the diameter of the largest tumor is mentioned

^b^Breast cancer in the ipsilateral or contralateral breast in patients with a personal history of breast cancer

^1^DCIS with micro-invasive growth

**Table 4:** Number of screens needed (NSN) for one additional mammography-only detected cancer

	Age group (years)	Number of breast cancers	Number of screens	Breast cancers detected by mammography only	NSN for mammography to detect breast cancer missed by MRI
Overall	< 40 years	26	1829	1	1829
	40–50 years	32	2050	2	1025
	50–60 years	45	1833	8	229
	≥ 60 years	22	841	2	421
* BRCA*	< 40 years	17	1113	0	N/A
	40–50 years	14	737	0	N/A
	50–60 years	26	568	3	189
	≥ 60 years	4	190	0	N/A
No *BRCA*	< 40 years	9	716	1	716
	40–50 years	18	1313	2	657
	50–60 years	19	1265	5	253
	≥ 60 years	18	651	2	326
Follow up	< 40 years	17	1112	1	1112
	40–50 years	20	1447	2	724
	50–60 years	28	1342	8	168
	≥ 60 years	11	626	2	313
* BRCA*	< 40 years	12	725	0	N/A
	40–50 years	9	554	0	N/A
	50–60 years	18	433	3	144
	≥ 60 years	0	152	0	N/A
No *BRCA*	< 40 years	5	387	1	387
	40–50 years	11	893	2	447
	50–60 years	10	909	5	182
	≥ 60 years	11	474	2	237

*MRI* magnetic resonance imaging, *N/A* not applicable, the first round was not shown in the table as no mammography-only cancers were detected in the first round

### Cancer yield

**Fig. 1:**
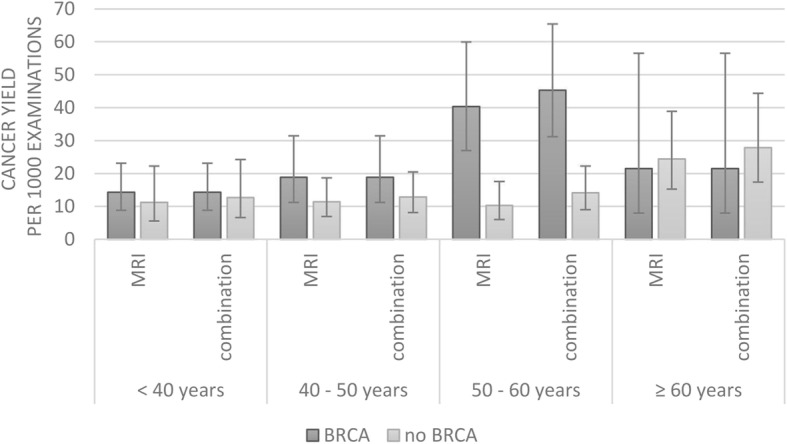
Cancer yield. Cancer yield in women with a *BRCA* mutation versus all others (family, personal, and others). The tag “combination” indicates the combination of mammography + magnetic resonance imaging (MRI)

**Fig. 2:**
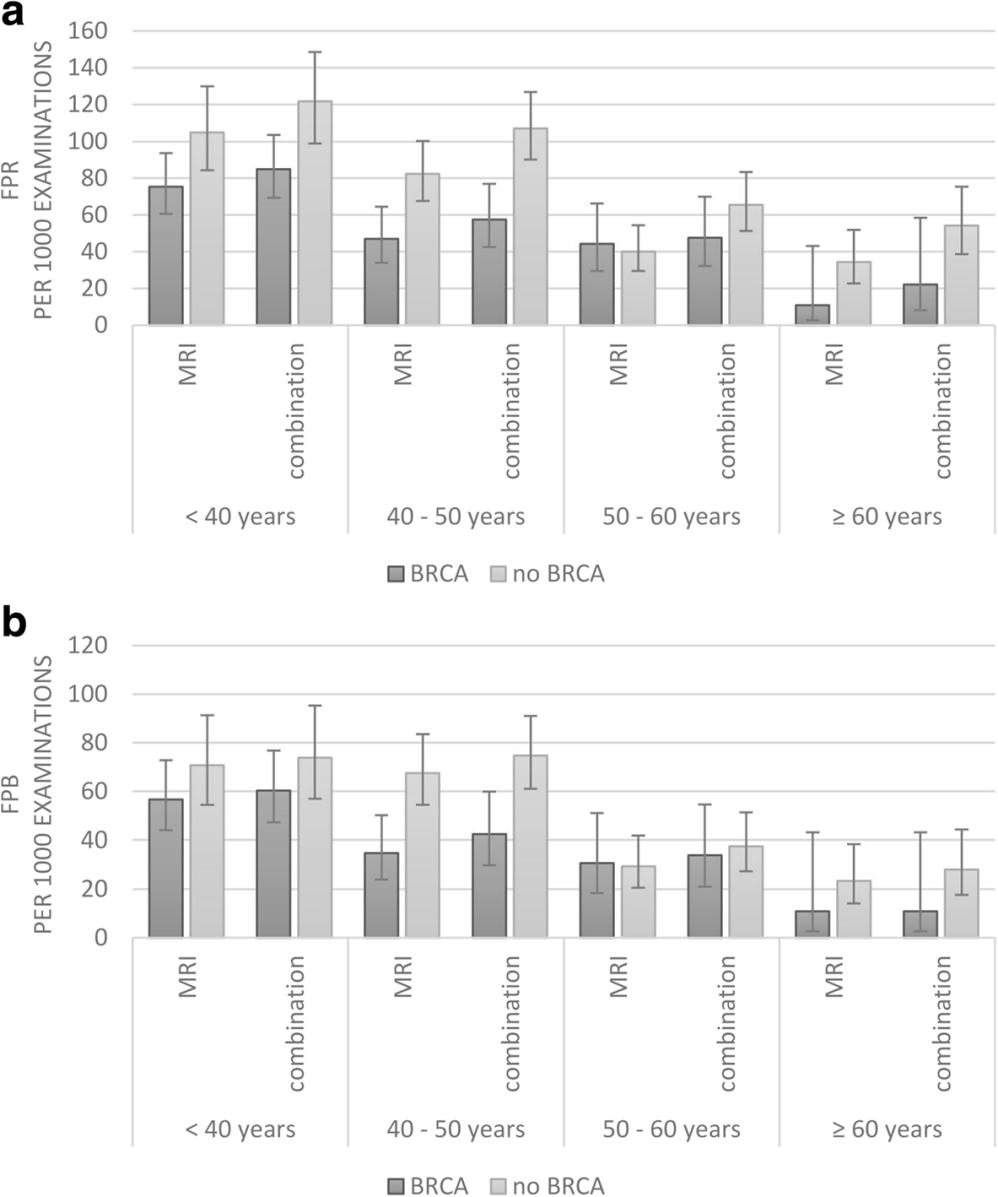
False positive rates. False positive rates for recall (FPR) (**a**) and for biopsy (FPB) (**b**) for women with a *BRCA* mutation versus all others (family, personal, and others). The tag “combination” indicates the combination of mammography + magnetic resonance imaging (MRI)

Cancer yield increased over time, with a peak at the 50–60 years age category (Fig. 1). The difference between cancers detected by MRI and the combination (mammography + MRI) seemed to increase with age (< 40 years, 0.47; 40–50 years, 0.93; 50–60 years, 4.26; ≥ 60 years, 2.93 per 1000 examinations), pointing to a possible increased added value of mammography in higher age categories (Fig. 2), which was the strongest in the 50–60 years categories both in the *BRCA* mutation carriers and others. The increase in breast cancer yield by the addition of mammography was not significant in any risk category (*p* ≥ 0.303). Table 5 summarizes cancer yield, FPR, and FPB.

**Table 5:** Cancer yield, FPR and FPB results for mammography (A), MRI (B), and the combination (C)

Age category	Risk category	Cancer yield^a^ (95% CI)	FPR^a^ (95% CI)	FPB^a^ (95% CI)
A. Mammography				
< 40 years	Overall	6.54 (3.73–11.46)	36.06 (27.57–47.03)	19.48 (13.73–27.58)
	*BRCA*	7.17 (3.61–14.18)	26.96 (18.07–40.03)	15.52 (9.03–26.57)
	No *BRCA*	5.61 (2.10–14.89)	49.62 (34.62–70.64)	24.94 (15.94–38.83)
40–50 years	Overall	6.35 (3.71–10.83)	40.44 (31.53–51.72)	24.23 (17.66–33.16)
	*BRCA*	6.78 (2.85–16.02)	18.90 (11.26–31.56)	12.21 (6.37–23.30)
	No *BRCA*	6.11 (3.08–12.06)	52.66 (39.80–69.38)	30.56 (21.36–43.55)
50–60 years	Overall	13.57 (9.21–19.94)	33.28 (24.94–44.29)	18.38 (12.60–26.75)
	*BRCA*	26.10 (15.93–42.47)	10.56 (4.75–23.31)	8.85 (3.67–21.15)
	No *BRCA*	7.89 (4.26–14.58)	43.06 (31.68–58.27)	22.16 (14.61–33.47)
≥ 60 years	Overall	17.72 (10.81–28.93)	25.82 (16.76–39.58)	10.88 (5.64–20.90)
	*BRCA*	21.48 (7.99–56.49)	10.69 (2.64–42.22)	0.00 (N/A)
	No *BRCA*	16.90 (9.54–29.77)	30.38 (19.27–47.57)	14.14 (7.31–27.15)
Overall	Overall	9.95 (7.80–12.69)	36.42 (31.42–42.19)	20.05 (16.49–24.37)
	*BRCA*	12.44 (8.75–17.65)	20.65 (15.32–27.77)	12.56 (8.50–18.51)
	No *BRCA*	8.33 (5.95–11.65)	47.08 (39.74–55.71)	24.95 (19.91–31.24)
B. MRI				
< 40 years	Overall	13.09 (8.80–19.44)	87.25 (74.68–101.71)	62.30 (51.89–74.63)
	*BRCA*	14.31 (8.81–23.16)	75.49 (60.59–93.70)	56.72 (44.03–72.79)
	No *BRCA*	11.22 (5.61–22.31)	104.96 (84.38–129.84)	70.70 (54.44–91.35)
40–50 years	Overall	14.06 (9.83–20.07)	70.45 (59.44–83.31)	55.95 (46.39–67.33)
	*BRCA*	18.86 (11.26–31.42)	47.03 (34.06–64.61)	34.73 (23.93–50.15)
	No *BRCA*	11.37 (6.91–18.66)	82.49 (67.63–100.27)	67.55 (54.44–83.53)
50–60 years	Overall	19.56 (14.16–26.96)	41.64 (32.53–53.16)	29.88 (22.29–39.94)
	*BRCA*	40.37 (27.00–59.96)	44.36 (29.42–66.36)	30.66 (18.27–51.02)
	No *BRCA*	10.25 (5.97–17.55)	40.08 (29.42–54.38)	29.41 (20.59–41.84)
≥ 60 years	Overall	23.56 (15.39–35.93)	29.04 (19.45–43.15)	20.49 (12.72–32.85)
	*BRCA*	21.48 (7.99–56.49)	10.85 (2.66–43.12)	10.85 (2.66–43.12)
	No *BRCA*	24.40 (15.23–38.87)	34.37 (22.63–51.89)	23.31 (14.04–38.45)
Overall	Overall	16.64 (13.81–20.04)	62.52 (56.37–69.29)	46.27 (41.11–52.06)
	*BRCA*	22.08 (17.03–28.60)	56.98 (48.07–67.42)	41.81 (34.35–50.80)
	No *BRCA*	13.10 (10.02–17.10)	66.13 (58.03–75.27)	49.21 (42.42–57.03)
C. Combination				
< 40 years	Overall	13.56 (9.24–20.10)	99.78 (86.35–115.04)	65.68 (54.96–78.31)
	*BRCA*	14.31 (8.81–23.16)	85.01 (69.45–103.66)	60.34 (47.29–76.70)
	No *BRCA*	12.65 (6.58–24.21)	121.64 (99.00–148.59)	73.85 (56.96–95.26)
40–50 years	Overall	15.02 (10.64–21.17)	89.74 (77.29–103.98)	63.26 (53.13–75.18)
	*BRCA*	18.86 (11.26–31.42)	57.42 (42.61–76.97)	42.39 (29.86–59.85)
	No *BRCA*	12.89 (8.10–20.48)	107.19 (90.27–126.84)	74.68 (61.11–90.97)
50–60 years	Overall	23.82 (17.82–31.76)	60.11 (48.79–73.85)	36.58 (28.05–47.59)
	*BRCA*	45.29 (31.14–65.43)	47.62 (32.20–69.90)	33.91 (20.91–54.54)
	No *BRCA*	14.16 (8.97–22.29)	65.53 (51.26–83.42)	37.51 (27.28–51.37)
≥ 60 years	Overall	26.49 (17.31–40.36)	46.86 (34.05–64.17)	24.16 (15.57–37.33)
	*BRCA*	21.48 (7.99–56.49)	22.05 (8.12–58.46)	10.85 (2.66–43.12)
	No *BRCA*	27.87 (17.39–44.38)	54.21 (38.71–75.43)	28.06 (17.67–44.30)
Overall	Overall	18.68 (15.65–22.27)	79.65 (72.70–87.21)	51.73 (46.28–57.78)
	*BRCA*	23.17 (18.00–29.79)	65.59 (56.09–76.55)	46.14 (38.31–55.47)
	No *BRCA*	15.74 (12.28–20.15)	89.14 (79.65–99.64)	55.39 (48.20–63.57)

^a^General estimating equations were used to calculate performance measures, correcting for multiple screening rounds within the same patient. All measurements are per 1000 examinations

*MRI* magnetic resonance imaging, *N/A* no cancers, recalls, or biopsies were found in this category and no 95% CI of this measure could be calculated, *95% CI* Wald 95% confidence intervals

### False positives

For FPRs, mammography added 103 FPRs on top of 112 FPRs based on both mammography and MRI, and 287 FPRs based on MRI alone. Overall, mammography significantly added to the FPRs (*p* = 0.001), especially in the group of women without a *BRCA* mutation (*p* = 0.001). The relative increase in the FPR due to mammography was greater in the higher age groups (< 40 years, 14%; 40–50 years, 27%; 50–60 years, 44%; ≥ 60 years, 61%, Fig. 2). This was significant in women without a *BRCA* mutation (*p* < 0.001). In total 35 FPBs were performed based on mammography alone. This did not lead to a significant increase in the overall FPBs (*p* = 0.013), or in any of the subcategories (*p* ≥ 0.323). Completely omitting mammography from the screening regimen would have led to a reduction of 21% (103/502) in FPRs and 11% (35/331) in FPBs.

## Discussion

This study evaluated the added value of mammography on top of MRI in a multimodal imaging screening program for women who are at intermediate or high risk of developing breast cancer in a single academic institute. The addition of mammography translated mostly to the detection of a small number of DCIS cases that were occult on MRI. However, five additional invasive carcinomas were also detected. The number of mammography screening examinations needed to detect an MRI occult cancer depended on age, and was very high in women under 40 years old. In addition, adding mammography led to a slight increase in false-positive recalls and biopsies.

Screening, with the aim of early detection of (pre-) malignant breast lesions to decrease breast cancer-related mortality, is a well-accepted risk-reducing strategy for most women at increased risk of developing breast cancer [24]. MRI is considered the most accurate imaging modality [10, 12, 25, 26]. Mammography is currently added to most screening regimens that include MRI to detect calcified breast lesions that may be visualized with mammography but not with MRI [27, 28]. In our study, 8 out of 13 cancers (62%) were MRI-occult DCIS that were detected based on microcalcifications on the mammogram. The five invasive cancers that were detected only with mammography, were also found because of microcalcifications. By mammography alone, only one invasive cancer (grade 3) was detected in a *BRCA* mutation carrier, at the age of 56 years. Our results are in line with the meta-analysis of Heijnsdijk et al. [29], who reported only one invasive cancer detected by mammography alone in *BRCA1* mutation carriers across four breast cancer screening trials of women at high risk of developing breast cancer. Obdeijn et al. [13] also reported little benefit of mammography screening in younger women with a *BRCA1* mutation. In their study, omitting mammography from the screening regimen would have led to two missed DCIS cases in women aged 50 and 67 years. Obdeijn et al. suggested to increase the starting age for mammography screening in women with *BRCA1* mutations to 40 years. Interestingly, in our study all cancers detected by mammography alone were detected in follow-up rounds, which might point to some increased value in higher age groups. It may also be partly explained by the fact that *BRCA* mutation carriers start with MRI alone, and only from the age of 30 years is mammography added. Our results suggest that the detection of MRI-occult breast cancers is very rare in all women younger than 40 years. Of 13 MRI-occult cancers (both DCIS and invasive cancers and both high and low grade), 10 were observed in women ≥ 50 years old in our population, which is in line with the results reported by Narayan et al. [30]. It should be noted that according to Vreemann et al. [31] 3 of the 13 MRI-occult cancers in this study were in retrospect visible on MRI, including 2 invasive ductal carcinoma (IDC) and 1 DCIS. In our study, raising the starting age of mammography to 40 years would have led to missing one invasive ductal cancer (high grade) in a woman with a positive family history of breast cancer but without a known *BRCA* mutation, and no DCIS would have been missed. In retrospect, this invasive cancer was one of the visible lesions on the MRI and was therefore not truly occult [31, 32]. Additionally, while in older women the additional detection of breast cancer increases with the addition of mammography, this is counterbalanced by an increase in false positive findings. These results are supported by the data of Phi and coworkers [33].

Other imaging modalities may be used to detect additional cancers on top of MRI. Unfortunately, handheld ultrasound or even automated breast ultrasound has been shown to be of limited value in a screening setting where MRI is available [10–12, 25, 34]. Digital breast tomosynthesis (DBT) has also been shown to increase the cancer detection rate and decrease the number of FPRs when compared to mammography alone in women at average risk [35, 36]. However, there is no consensus on the added value of DBT when breast MRI is available [37]. Therefore, current guidelines only include mammography. The gain in sensitivity with mammography seems to come mostly from the detection of lesions presenting with calcifications. DBT appears to be of relatively equal value to mammography for this purpose, but at a higher dose [38, 39]. Since younger women at high risk and in particular *BRCA* mutation carriers have been shown to be more susceptible to developing radiation-induced cancers [17, 18], replacing mammography with DBT might not be beneficial for women screened with MRI. Berrington de Gonzalez et al. [17] reported no net benefit of mammography surveillance before the age of 35 years in women with a *BRCA* mutation and recommended to limit the radiation dose by raising the age for undergoing mammography. Our results indicate that raising the age limit of supplemental mammography screening to the age of 40 years should be considered, not only for *BRCA* germline mutation carriers, but for all women at increased risk of developing breast cancer.

A further reason for this recommendation is that population-based mammography screening programs have been criticized because of overdiagnosis and overtreatment of non-fatal breast disease detected during screening [40]. Overdiagnosis, defined as the detection of a breast cancer at screening that would have never been identified clinically in the lifetime of the woman, has been reported as between 1 and 10% [41]. Our results suggest that adding mammography screening to breast MRI may contribute to overdiagnosis because of the preferential detection of relatively indolent (pre-) malignant subtypes such as low-grade calcified ductal in situ carcinoma as described in a previous study [20]. These cancers might be biologically irrelevant compared to invasive and in situ cancers detected with MRI that tend to be of higher grade and are usually detected at an earlier stage [10, 37]. However, this is not evident from our data.

Our study has some limitations. It is a single-center study in a tertiary referral center with a large, high-risk screening program that might not be fully generalizable to the whole breast imaging community. In addition, due to the retrospective nature of the study, some of the MRI and mammography examinations were evaluated simultaneously, which might affect the screening outcomes either positively or negatively. Breast density and background parenchymal enhancement were often not reported and therefore not used in this analysis. While the study describes a long time-span, the absolute number of cancers detected is still small, which might lead to underpowered results. Therefore, more studies are required to confirm our findings.

## Conclusions

In conclusion, mammography does not appear to significantly add to cancer yield, albeit our results must be interpreted with the relatively small number of cancers in our study. In *BRCA* mutation carriers the added cancer detection with mammography is even less than for women without *BRCA* mutation. Especially in younger women, the number of mammography screens needed to detect one additional cancer is very high, and increasing the starting age for mammography (if at all) seems safe to maximize the benefits of MRI screening. In higher age groups mammography does add to the detection rates, but also leads to an increase in FPR and FPB.
